# m6A Regulatory Gene-Mediated Methylation Modification in Glioma Survival Prediction

**DOI:** 10.3389/fgene.2022.873764

**Published:** 2022-04-26

**Authors:** Guiyun Zhang, Ping Zheng, Yisong Lv, Zhonghua Shi, Fei Shi

**Affiliations:** ^1^ Department of Neurovascular Intervention, Clinical Center of Neuroscience, Shanghai General Hospital, Shanghai Jiao Tong University School of Medicine, Shanghai, China; ^2^ Department of Neurosurgery, Shanghai Pudong New Area People’s Hospital, Shanghai, China; ^3^ School of Continuing Education, Shanghai Jiao Tong University, Shanghai, China; ^4^ Department of Neurosurgery, 904th Hospital of PLA, Wuxi, China

**Keywords:** glioblastoma, low grade glioma, TCGA, M6A, immune infiltration

## Abstract

The median survival of patients with gliomas is relatively short. To investigate the epigenetic mechanisms associated with poor survival, we analyzed publicly available datasets from patients with glioma. This analysis revealed 12 prognosis-related m6A regulatory genes that may be responsible for poor prognosis. These genes may be involved in genomic changes inherent to oxidative phosphorylation, adipogenesis, hedgehog signaling, and Myc signaling. We reconstructed a risk model with univariate and multivariate Cox analyses and identified older age and the m6A risk score as independent risk factors for predicting the prognosis of glioma patients, which is associated with glioma immune infiltration. In conclusion, m6A regulatory genes may serve as both reliable biomarkers and potential targets to increase the chance of survival of patients with glioma.

## Introduction

Gliomas are the most common and malignant brain tumors. Despite the progress made in the diagnosis and treatment of brain tumors, the overall survival rate for glioma is quite low (Wijethilake et al., 2021). Less than 10% of patients responds to standard therapy and lives longer than 2 years (Olgun et al., 2021). Additionally, the prognosis of individual patients with glioma is difficult to predict because few clinical biomarkers or parameters are available to reflect disease progression and neurological outcomes. Although a series of functional gene sets have been identified, the exact roles of these clusters remain to be elucidated (He et al., 2020; Wei et al., 2020; Zhang et al., 2021). Thus, a better understanding of the molecular mechanisms of glioma, including its genetic background and prognosis-related factors, is essential for the diagnosis and treatment of this malignant disease.

Epigenetic modifications of DNA and RNA play a critical role in brain function (Dong and Cui, 2019, 2020; Deng et al., 2020). Among these, N6-methyladenosine (m6A) methylation is of particular interest as it occurs in more than one hundred thousand coding and non-coding RNAs(Chen et al., 2019; Haixu Wang et al., 2021; Liu and Su, 2021). However, the exact role of m6A-related genes and their expression profiles in gliomas remain elusive (Cui et al., 2017). Next-generation sequencing has allowed to obtain the genetic profile in mRNA and m6A genes present in The Cancer Genome Atlas—TCGA (Lauber et al., 2018; Sharma et al., 2019; Deng et al., 2021). However, few bioinformatics studies have investigated the correlation between coding and non-coding RNAs and m6A marker genes.

In this study, we first profiled m6A-related genes in glioma and constructed a risk prediction model based on these genes to investigate the functional enrichment and outcome prediction ability. Moreover, we investigated the relationship between high- and low-risk scores of genomic changes and immune infiltration in glioma patients.

## Materials and Methods

### Data Download

The expression profiling data (FPKM) of patients with glioblastoma multiforme (GBM) and lower-grade glioma (LGG) were downloaded from TCGA GDC official website (https://portal.gdc.cancer.gov/), and the FPKM was then converted to the TPM value. The clinical data included age, sex, and survival prognosis, and after deleting the missing information, 638 tumor tissues and five normal tissues were obtained. The copy number variation (CNV) of glioma patients was also downloaded, and the RCircos package (Zhang et al., 2013) was used to map the genetic copy number variation in 23 pairs of chromosomes. After selecting “Masked Somatic Mutation” as the somatic mutation data, we used the maftools package (Mayakonda et al., 2018) to visualize somatic mutations and obtain the tumor mutation burden (TMB) of each patient. In addition, the sequencing results of 970 glioma patients were downloaded from the CGGA database, and the sequencing results of 2,642 normal brain tissues were downloaded from the GTEx database and converted into TPM values. Finally, 2,647 normal brain tissues and 1,608 glioma tissues were sequenced.

### Construction of a Risk Model Based on m6A-Related Genes

To analyze the expression of m6A-related genes in glioma, we first analyzed the differential expression of m6A-related genes between glioma and normal tissues, the correlation of gene expression, and its influence on the prognosis of patients with glioma. Subsequently, using the expression profiling of both TCGA-glioma data and CGGA-glioma data, the m6A-related genes were incorporated into the risk model, and the least absolute shrinkage and selection operator (LASSO) algorithm was used to perform dimensionality reduction analysis and obtain prognosis-related genes. The normalized gene expression value weighted by the penalty coefficient obtained by LASSO Cox analysis established a risk score formula, and the median value of the risk score was used to divide the patients into high- and low-risk groups.

$$\text{risk}Score\;=\sum_{i}Coefficient\;\left(hub\;gene_{i}\right)*mRNA\;Expression\;\left(hub\;gene_{i}\right)\;$$

### Functional Enrichment Analysis and Gene Set Enrichment Analysis (GSEA)

Gene ontology (GO) analysis is a common method for large-scale functional enrichment research, including biological processes (BP), molecular functions (MF), and cellular components (CC). The Kyoto Encyclopedia of Genes and Genomes (KEGG) is a widely used database that stores information on genomes, biological pathways, diseases, and drugs. The R clusterProfiler package (Wu et al., 2021) was used to perform GO annotation and KEGG pathway enrichment analysis for the signature genes. The critical value of FDR <0.05 is considered statistically significant.

To study the differences in biological processes based on the gene expression profiling data of glioma patients, we performed gene set enrichment analysis (GSEA). GSEA calculates the statistical difference between two biological states in a specific gene set (Canzler and Hackermüller, 2020) and is usually used to estimate the changes in pathway and biological process activity in the dataset. The “h.all.v7.2. symbols.gmt” gene set was downloaded from the MSigDB database (Liberzon et al., 2015) for the analysis. Statistical significance was set at an adjusted *p*-value of less than 0.05.

### Assessment of the Biological Characteristics of Patients Between Risk Groups

We used the GSVA method (Ferreira et al., 2021) to analyze the correlation between different groups and biological processes. Mariathasan et al. (2018) constructed a set of genes to store those related to certain biological processes, including 1) immune checkpoints, 2) antigen processing, 3) CD8^+^ T cell characteristics, 4) epithelial–mesenchymal transition (EMT) markers, including EMT1, EMT2, and EMT3, 5) angiogenesis characteristics, 6) pan-fibroblast TGF-β response characteristics (Pan-FTBRS), 7) WNT characteristics, 8) DNA damage repair, 9) mismatch repair, (10) nucleotide excision repair, 11) DNA replication, and 12) antigen processing and presentation. The gene sets were downloaded according to different biological characteristics to calculate the enrichment scores corresponding to the patients and compare the differences between the two groups.

### Analysis of m6A-Related Clusters in DEGs

The R limma package (Ritchie et al., 2015) was used to analyze the differentially expressed genes (DEGs) between the high-and low-risk glioma patient groups to determine the genes associated with the m6A risk model. The DEGs were defined as those with an absolute value of log(fold change) > 1.5 and an FDR <0.01. The tumor was divided into different groups based on the Euclidean distance, and named as genecluster using the hierarchical clustering method, where the R ConsensuClusterPlus package (Wilkerson and Hayes, 2010) was used to determine the number of clusters in the dataset, and repeated 1,000 times to ensure the stability of classification. At the same time, based on the expression profile of specific genes, they were divided into two groups: signature genes A and B.

### Dimensionality Reduction and Calculation of the m6A Score

First, according to the DEG value, unsupervised clustering classified the patients in TCGA. According to the Boruta algorithm, the m6A Signature-A and B gene clusters are reduced in dimensionality, and PC1 is extracted as a score using the PCA algorithm. Finally, we applied a method similar to that of the gene expression grade index to define the ICI score of each patient:

$$m6A\;score\;=\;\sum_{i}PC1A-\sum_{i}PC1B$$

### Identification and Correlation Analysis of Tumor Immune Infiltrating Cells

To further quantify the proportion of different immune cells in the glioma sample, we used a single-sample GSEA algorithm to distinguish human immune cell phenotypes in the tumor immune microenvironment (TME) with high sensitivity and specificity. The ssGSEA algorithm was used to quantify the relative content of tumor-infiltrating immune cells in patients with glioma (Minjie Wang et al., 2021). The algorithm identified 28 genes for marking different tumor-infiltrating immune cell types through the research of Bindea et al. (2013). The gene set contained various human immune cell subtypes, including CD8^+^ T cells, dendritic cells, macrophages, and regulatory T cells. The enrichment score calculated by ssGSEA analysis with the R GSVA package (Minjie Wang et al., 2021) can be used to represent the infiltration level of each immune cell type in each sample.

The ESTIMATE package (Yoshihara et al., 2013) is used to evaluate the immune activity of the tumors. ESTIMATE analysis quantifies the immune activity (immune infiltration level) in the tumor sample based on its gene expression profile and obtains an immune score for each tumor sample. This can be used to compare the differences in immune infiltration between the high- and low-risk groups of GLIOMA patients.

### Copy Number Variation Analysis

To analyze the copy number changes in different risk score groups of TCGA-glioma patients, we used R’s TCGAbiolinks package to download the masked copy number segment data of patients. GISTIC 2.0 analysis was performed on the downloaded CNV fragments using GenePattern5. During the GISTIC 2.0 analysis, the default settings were used, except for a few parameters (for example, the confidence level was 0.99; the X chromosome was not excluded before the analysis). Finally, we used R’s Maftools package to visualize the analysis results.

### Anti-cancer Drug Sensitivity Analysis

The Genomics of Drug Sensitivity in Cancer (GDSC; https://www.cancerrxgene.org/) is a public database for molecular cancer therapy and mutation exploration (Yang et al., 2013). We used R’s pRRophetic package (Geeleher et al., 2014) to download cell line gene mutation data and IC_50_ values of different anti-cancer drugs. We then analyzed the correlation between patients with high- and low-risk scores and the sensitivity to different anti-cancer drugs.

### Building a Clinical Prediction Model Based on the m6A Risk Model

Univariate and multivariate Cox analyses were used to analyze the risk score combined with the patient’s clinicopathological characteristics to predict the overall survival (OS) to prove that the risk score combined with clinicopathological characteristics can evaluate the prognosis for the individual patient. Subsequently, the risk scoring model combined with clinicopathological characteristics was selected to construct a clinical prediction nomogram. Harrell’s consistency index (C-index) was measured to quantify the discrimination performance. A calibration curve was generated to evaluate the performance of the nomogram by comparing its predicted value with the actual survival rate.

### Immunohistochemical Gene Validation

To validate m6A gene expression, we performed immunohistochemistry (IHC) on surgical human glioma samples. Using available antibodies, we selected three genes of interest: IGF2BP3 (14642-1-AP, Proteintech, China), RBM15B (22249-1-AP, Proteintech, China), and RBM15 (10587-1-AP, Proteintech, China). The comparison was performed between the glioma sample and the para-tumor area, performed under the approval of local medical ethics (No.2020SQ119) in Shanghai General Hospital, affiliated to Shanghai Jiaotong University.

### Statistical Analysis

All data processing and analyses were performed using the R software (version 3.6.2). To compare two groups of continuous variables, the statistical significance of normally distributed variables was estimated using the independent Student’s t-test, and the differences between non-normally distributed variables were analyzed using the Mann–Whitney *U* test (i.e., Wilcoxon rank sum test). Chi-square or Fisher’s exact tests were used to compare and analyze the statistical differences between the two groups of categorical variables. Pearson correlation analysis calculated the correlation coefficients between different gene sets. The survival package in R was used for survival analysis. The Kaplan–Meier survival curve was used to show the survival difference. The log-rank test was employed to determine the significance of different survival times between the two groups. Univariate and multivariate Cox analyses were used to determine independent prognostic factors. All statistical *p*-values were two-sided, and statistical significance was set at *p* < 0.05.

## Results

### The Expression and Mutation Profile of m6A-Related Genes in Glioma Patients

**FIGURE 1:**
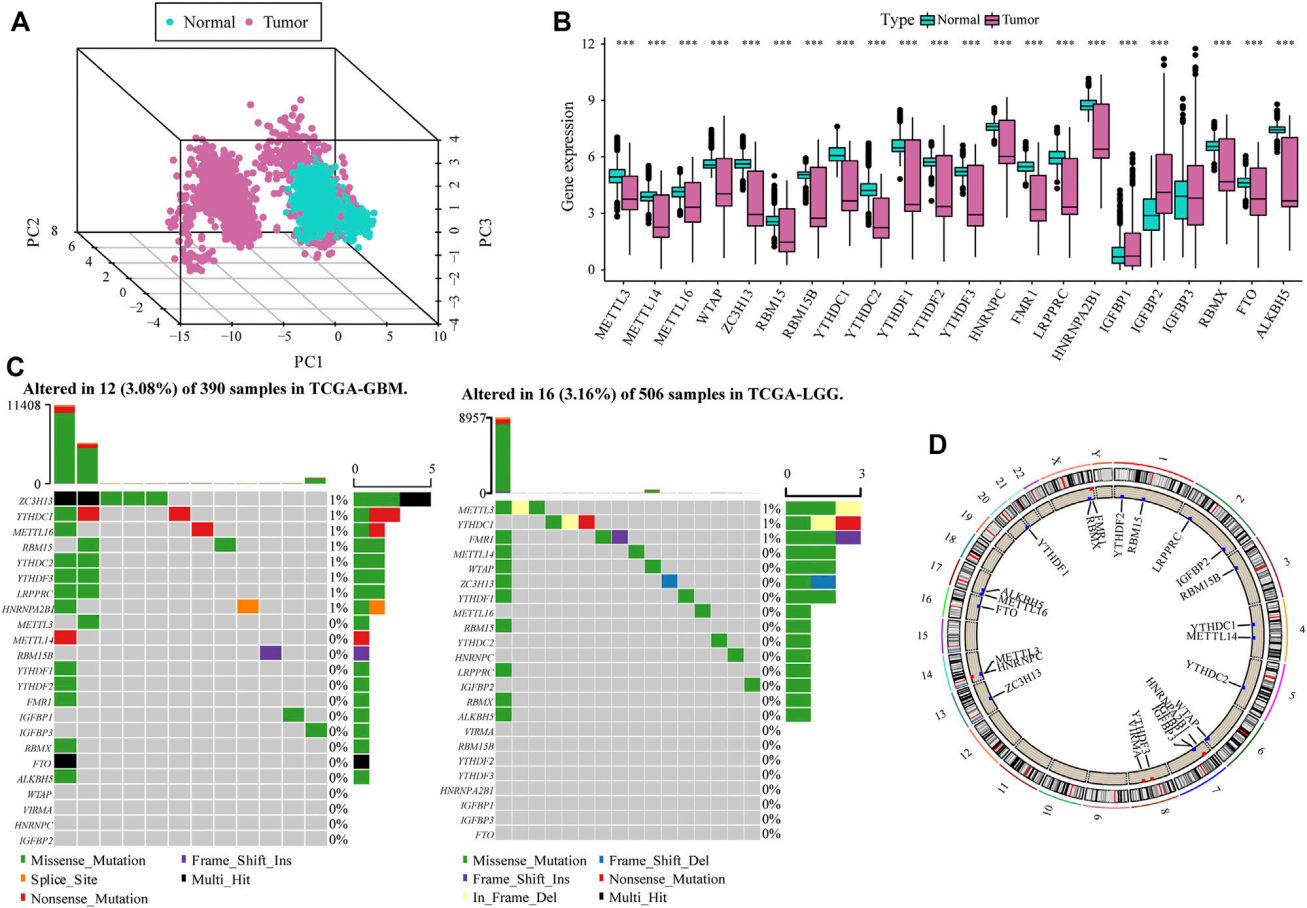
Overall m6A-related gene expression in glioma patients. **(A)** PCA shows that there are certain differences in the overall levels of m6A-related genes in glioma and normal brain tissue in the The Cancer Genome Atlas (TCGA), CGGA, and GTEx datasets; **(B)** Most m6A-related genes were expressed differently in glioma tissue compared with the normal brain tissue; **(C)** m6A-related gene mutation map in TCGA-glioma patients. The samples are sorted according to the burden of somatic non-synonymous mutations, and the genes are sorted by mutation frequency. Different colors indicate different mutation types; the upper section of the legend shows mutation load; **(D)** Differential copy number variation of m6A-related genes on 23 chromosomes in TCGA glioma data.

To analyze the overall expression of m6A-related genes in patients with glioma, we analyzed genomic mutations and mRNA expression, including gene expression levels, single nucleotide polymorphisms, and copy number variations. First, we conducted a comprehensive analysis of the expression in gliomas and normal brain tissues in TCGA, CGGA, and GTEx databases and used the de-batch method. PCA results showed that the characteristics of m6A-related genes differed between glioma and normal brain tissues (Figure 1A). Subsequently, the differential analysis showed that, between glioma and normal brain tissue, a variety of m6A-related genes were differentially expressed, including *METTL14*, *METTL16*, *ZC3H13*, *YTHDC1*, *YTHDC2*, *YTHDF2* (Zhang et al., 2017; Dixit et al., 2021; Fang et al., 2021)etc. (Figure 1B).

The results of single nucleotide polymorphism (SNP) analysis showed that among GBM samples, 12 had single nucleotide mutations in m6A-related genes, among which the mutation rate of the *ZC3HI3* gene was the highest. In contrast, in the LGG samples, 16 had single nucleotide mutations in m6A-related genes, and the mutation rate of *METTL3* was the highest (Figure 1C). Moreover, studies on the frequency of CNV changes have shown that m6A-related gene changes in CNV levels in glioma patients are common, and most of them are concentrated on copy number loss (Figure 1D).

### Construction of the m6A Expression Risk Model and Prognostic Analysis

**FIGURE 2:**
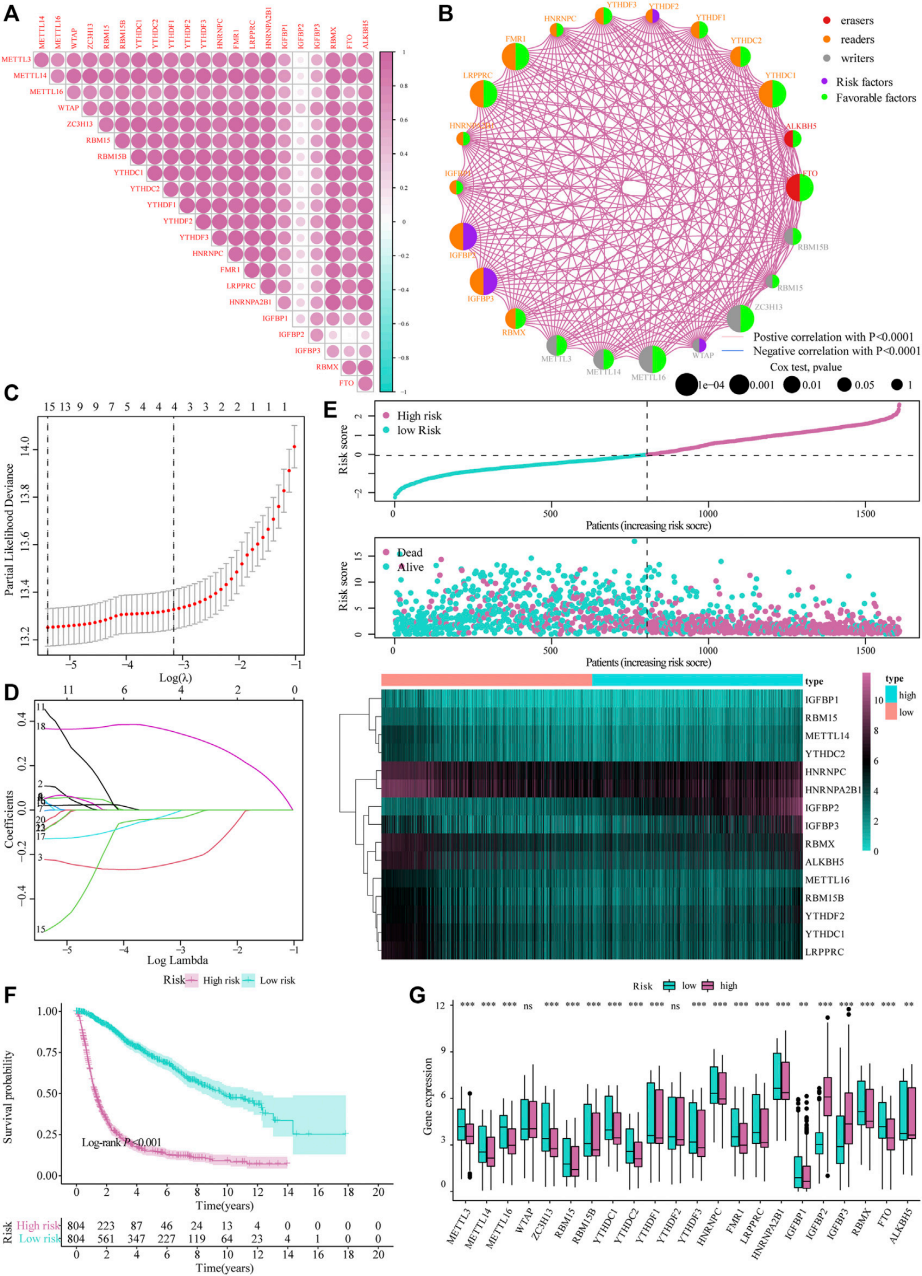
Construction of the m6A risk scoring model. **(A)** Correlation analysis of m6A-related gene expression in glioma; **(B)** Expression and interaction of m6A-related genes in glioma patients. The size of each cell represents the impact of each gene on the patient’s survival status, and the log-rank test was used for analysis. Half of the color of the circle represents the grouping of m6A-related genes, and the other half represents the impact on the prognosis. Among them, m6A-related gene groups: Erasers, red; Readers, orange; Writers, gray. At the same time, purple represents risk factors in the impact on prognosis, and the green represents protective factors. The lines connecting m6A-related genes represent the interactions between genes. The thickness of the line represents the correlation strength estimated by Spearman correlation analysis, red the negative correlations, and blue the positive; **(C,D)** LASSO Cox analysis identified 15 genes most relevant to the OS in the TCGA dataset; **(E)** The risk score distribution of glioma patients, the patient’s survival status and the heat map of characteristic gene expression; **(F)** Kaplan–Meier curve to assess risk score impact on the overall survival rate of glioma patients; **(G)** Differential expression of m6A-related genes at high and low m6A risks.

The heat map resulting from Pearson’s analysis revealed a positive correlation between m6A-related gene expression and glioma tissue (TCGA dataset) (Figure 2A). The detailed number of each coefficient is displayed in Supplementary data. We further analyzed the influence of m6A-related genes on the prognosis of patients with glioma in TCGA and CGGA databases. The gene network depicts the interaction of m6A-related genes in glioma and their impact on the overall survival of glioma patients (Figure 2B).

Subsequently, we integrated the expression of m6A-related genes to construct a risk-scoring system to quantify the impact of m6A-related genes on the prognosis of each glioma patient. First, m6A-related genes were included in the LASSO Cox analysis, and 15 genes with the best prognostic value were obtained (Figures 2C,D). Based on the penalty coefficients of important characteristic genes calculated by LASSO Cox analysis, the gene expression and the corresponding coefficients were multiplied, and the final risk score of each sample was calculated. The distribution of risk scores, survival status, and expression patterns of the feature genes is shown in Figure 2E. Kaplan-Meier analysis showed that the overall survival (OS) of patients with high-risk scores was relatively poor (log-rank *p* < 0.001; Figure 2F). At the same time, the differential analysis results showed significant differences in the expression of m6A-related genes between the high- and low-risk models (Figure 2G).

**FIGURE 3:**
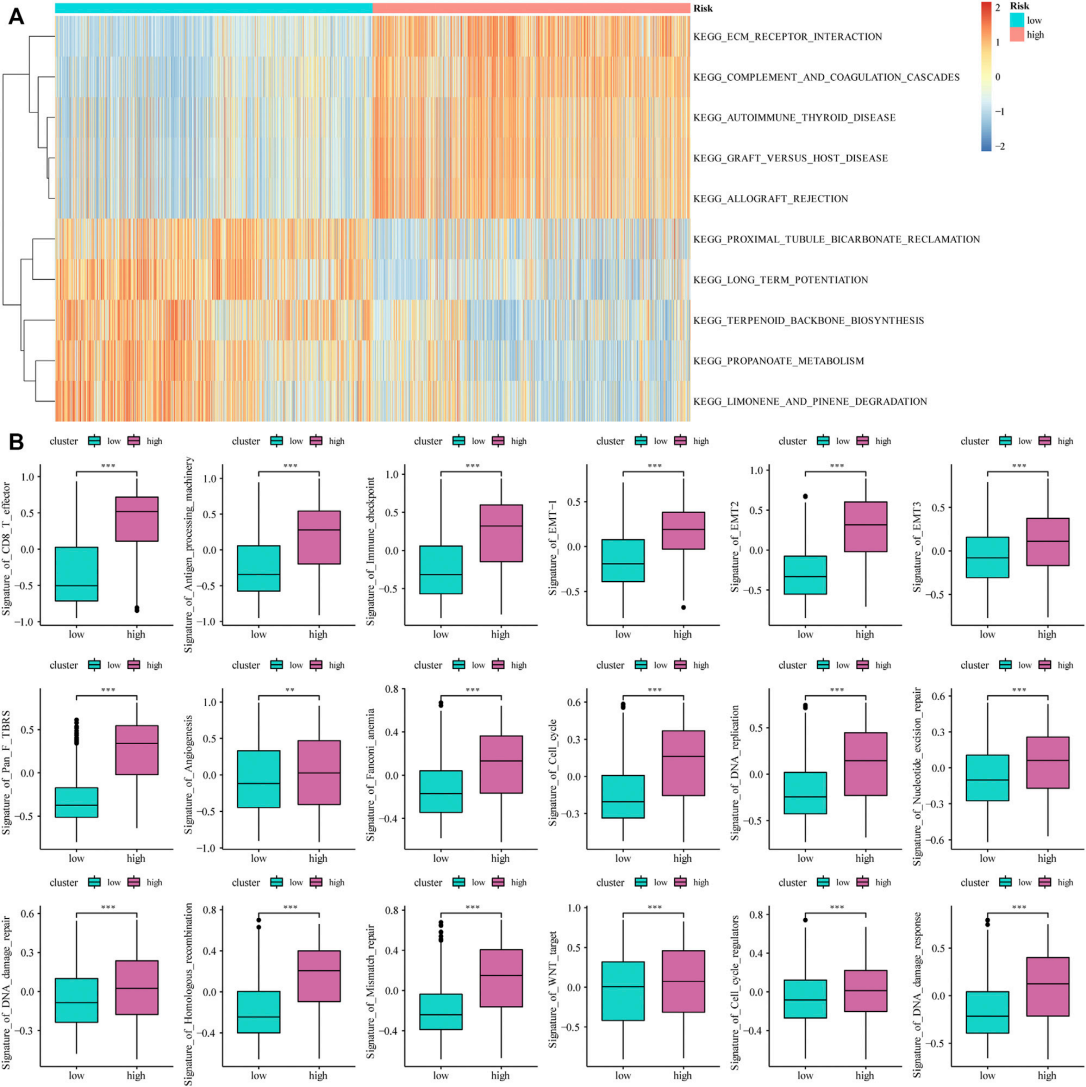
The influence of m6A risk model on different biological characteristics. **(A)** Based on the gene expression of glioma patients, we performed GSVA on high- and low-risk groups, and used heat maps to show related pathways with significant differential enrichment; **(B)** Different pathway (immune-related features, mismatches and clinical characteristics) enrichment in the high and low risk score groups, where the thick line represents the median value, and the bottom and top of the box are the 25th and 75th percentiles (interquartile range) (*p < 0.05, **p < 0.01, ***p < 0.001).

Based on the median value of the m6A risk score of glioma patients, we placed the patients into the high- or low-risk group and assessed the changes in biological function between the two groups. The GSVA method was used to determine the enrichment scores of these patients, and heat maps were used to show the relevant signaling pathways and analyze their variations in the two groups (Figure 3A). In addition, the results showed that there were significant differences in the enrichment of certain related biological pathways, such as CD8^+^ T cell effectors, immune checkpoints, EMT pathways, angiogenesis, and others (*p* < 0.05; Figure 3B).

### Construction of Genetic Characteristics of Glioma Patients Based on the m6A Risk Model

**FIGURE 4:**
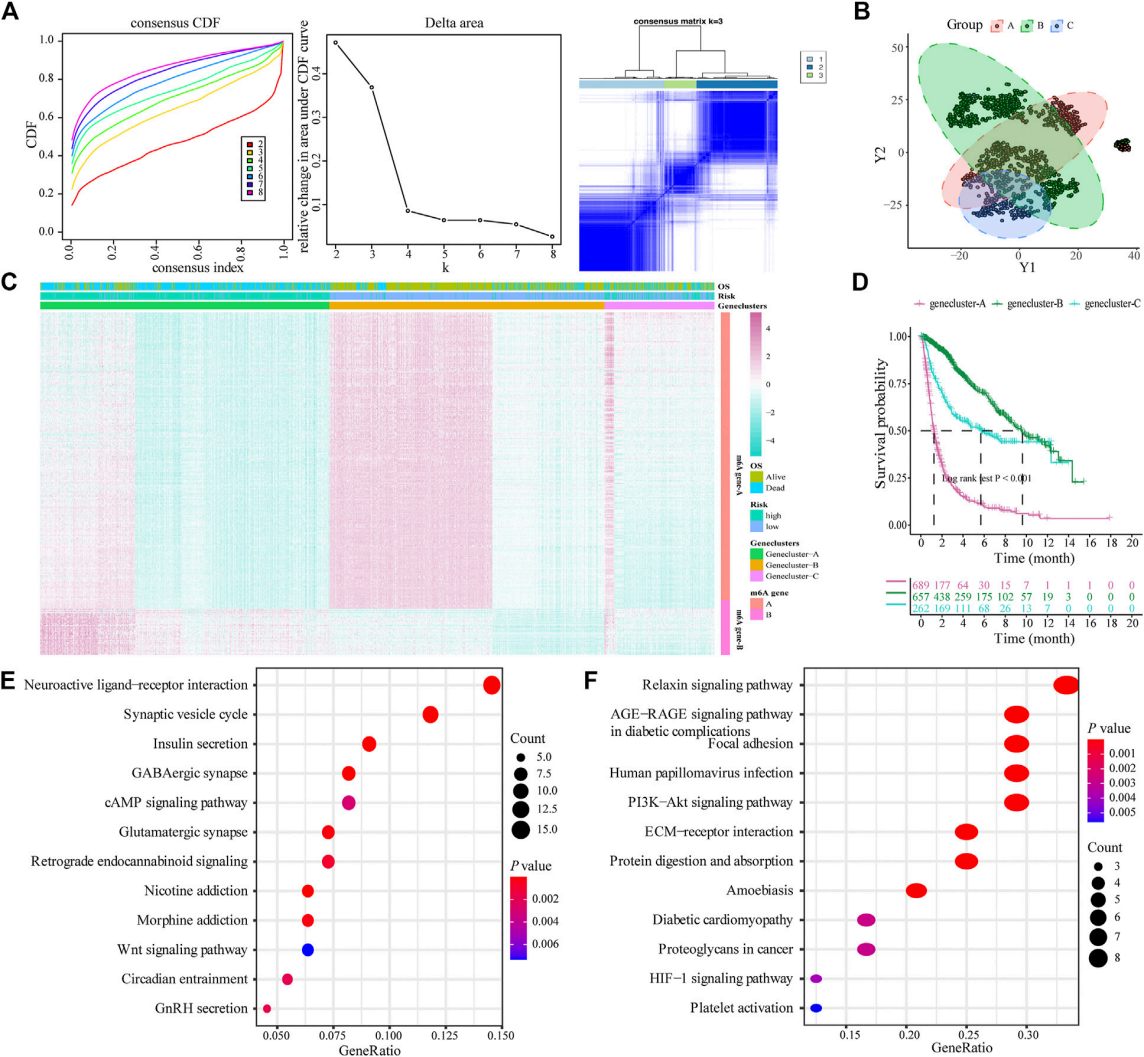
Construction and functional annotation of an m6A gene feature model of patients with glioma. **(A)** Based on the expression characteristics of differentially expressed genes between high and low m6A risk score groups, unsupervised analysis and hierarchical clustering were performed, and patients were divided into three categories, called genecluster-A, -B, and -C; **(B)** tSNE analysis showed differential gene expression in genecluster-A, -B, and -C; **(C)** Heat map shows the expression levels of characteristic genes among the three Geneclusters; **(D)** Survival analysis shows the different prognosis among genecluster groups, among which the prognosis of patients in the genecluster-A group is the worst; **(E)** The Kyoto Encyclopedia of Genes and Genomes (KEGG) analysis shows that Signature gene-A is closely related to pathways such as synaptic vesicle cycle, insulin secretion, nicotine addiction, and GABAergic synapses; **(F)** KEGG analysis shows that Signature gene-B is closely related to the relaxin signaling pathway, AGE-RAGE signaling pathway in diabetic complications, ECM-receptor interaction, and protein digestion and absorption pathways.

The limma package was used to analyze DEGs between different risk models, and 443 genes were obtained to determine the potential biological characteristics of different m6A-related phenotypes. Subsequently, based on the expression of DEGs, unsupervised clustering was used to divide glioma patients into three subtypes: genecluster-A, -B, and -C (Figure 4A). At the same time, tSNE analysis showed certain differences in gene expression levels between genes A, B, and C (Figure 4B). The heat map shows the gene expression characteristics of the three genotypes (Figure 4C). Meanwhile, the survival analysis results showed that there were significant differences in the prognosis of patients with the three different genotypes, among which the patients in the Genecluster-A group had the worst prognosis (log-rank *p* < 0.001; Figure 4D).

According to the expression and correlation of DEGs in these groups, the genes were divided into m6A signature-A and m6A signature-B. There were 268 m6A signature-A and 51 m6A signature-B gene sets. To explore the differences in biological functions between the two groups, we conducted a functional enrichment analysis. KEGG enrichment analysis showed that signature genes A and B showed different, unique biological processes (Tables 1, 2). m6A gene-A is involved in the synaptic vesicle cycle, insulin secretion, nicotine addiction, and GABAergic synapse pathways (Figure 4E), while the gene set overexpressing signature gene-B mainly manifests as the relaxin signaling pathway, AGE-RAGE signaling pathway in diabetic complications, ECM-receptor interaction, and protein digestion and absorption pathways (Figure 4F).

**TABLE 1:** KEGG analysis for m6A Signature gene-A.

Pathway ID	Pathway description	Count in gene Set	*p* Value
hsa04721	Synaptic vesicle cycle	13	2.80E-11
hsa04911	Insulin secretion	10	2.14E-07
hsa05033	Nicotine addiction	7	9.16E-07
hsa04727	GABAergic synapse	9	2.94E-06
hsa04080	Neuroactive ligand-receptor interaction	16	1.32E-05
hsa04724	Glutamatergic synapse	8	0.000151
hsa05032	Morphine addiction	7	0.000225
hsa04723	Retrograde endocannabinoid signaling	8	0.000884
hsa04929	GnRH secretion	5	0.001713

**TABLE 2:** KEGG analysis for m6A Signature gene-B.

Pathway ID	Pathway description	Count in gene Set	*p*-Value
hsa04926	Relaxin signaling pathway	8	2.00E-09
hsa04933	AGE-RAGE signaling pathway in diabetic complications	7	1.04E-08
hsa04512	ECM-receptor interaction	6	1.61E-07
hsa04974	Protein digestion and absorption	6	4.12E-07
hsa04510	Focal adhesion	7	1.28E-06
hsa05146	Amoebiasis	5	1.02E-05
hsa05165	Human papillomavirus infection	7	3.42E-05
hsa04151	PI3K-Akt signaling pathway	7	5.27E-05
hsa05415	Diabetic cardiomyopathy	4	0.002762

### Construction of a Prognostic-Related m6A Feature Model Based on m6A Gene Signature

**FIGURE 5:**
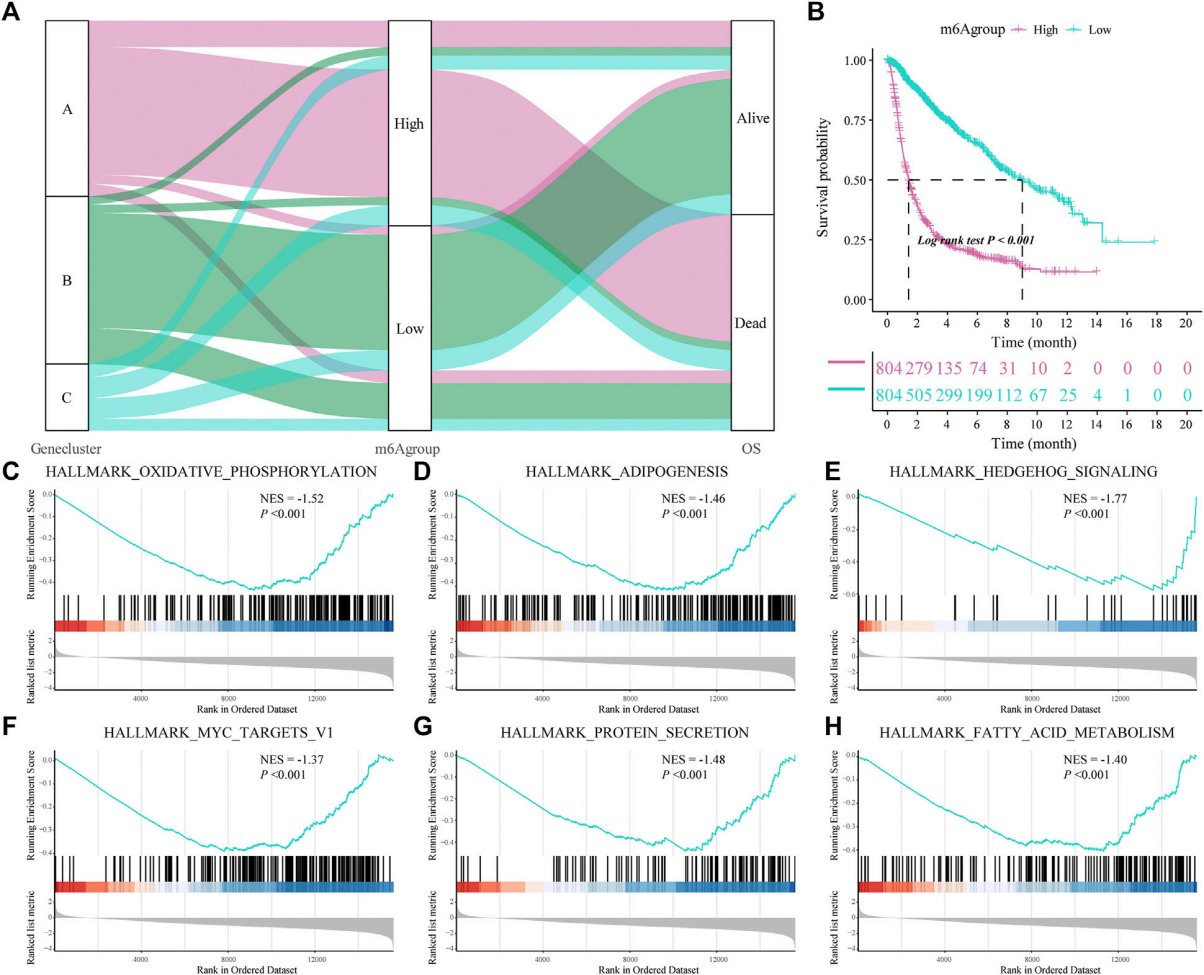
Construction of a prognostic-related m6A characteristic model and regulation of biological processes. **(A)** The sankey diagram shows the correlation between gene clusters, prognostic-related m6A features (m6Agroup), and patient prognostic status (OS); **(B)** Survival analysis shows that the prognostic-related m6A feature model can better predict the overall survival rate of glioma patients (Log-rank *p* < 0.001); **(C–H)** gene-set enrichment analysis (GSEA) of high- and low-risk patients, the representative gene set downloaded from the MSigDB database, with 1,000 repetitions for each run; GSEA results show that enriched genes of glioma patients in the low m6A group are closely related to hallmark oxidative phosphorylation, adipogenesis, hedgehog signaling and MYC Target V1.

We constructed a new prognostic-related risk-scoring system to better predict the impact of m6A features on patient prognosis. According to the expression of m6A signature genes A and B in glioma patients, principal component analysis was used to calculate the corresponding PCA1 of each patient, and the corresponding m6A score was obtained by subtraction and named m6A group. Similarly, based on the median score of the prognostic models, patients were divided into high- and low-risk groups. The Sankey diagram shows the correspondence between the gene cluster corresponding to each glioma patient, prognostic model of the m6A group, and patient survival status (Figure 5A). At the same time, the results of the survival analysis showed that the prognostic score model could predict well the OS of glioma patients (log-rank *p* < 0.001; Figure 5B).

Subsequently, we analyzed the biological effects of the high and low m6A groups. GSEA showed that hallmark oxidative phosphorylation, adipogenesis, hedgehog signaling and MYC target V1 were significantly enriched in glioma patients in the low m6A group (Table 3; Figures 5C–H).

**TABLE 3:** GSEA results.

Name	Size	Enrichment score	NES	*p*-Value	Leading edge
HALLMARK_OXIDATIVE_PHOSPHORYLATION	180	−0.43632	−1.5241	8.13E-07	tags = 71%
					list = 41%
					signal = 42%
HALLMARK_ADIPOGENESIS	186	−0.4171	−1.45876	6.53E-06	tags = 59%
					list = 38%
					signal = 37%
HALLMARK_HEDGEHOG_SIGNALING	34	−0.57873	−1.76828	5.87E-05	tags = 47%
					list = 13%
					signal = 41%
HALLMARK_MYC_TARGETS_V1	192	−0.39081	−1.36787	0.000129	tags = 78%
					list = 50%
					signal = 39%
HALLMARK_PROTEIN_SECRETION	95	−0.44101	−1.48158	0.000155	tags = 60%
					list = 32%
					signal = 41%
HALLMARK_FATTY_ACID_METABOLISM	146	−0.40521	−1.39875	0.000255	tags = 47%
					list = 27%
					signal = 34%
HALLMARK_MITOTIC_SPINDLE	197	−0.3749	−1.31414	0.000717	tags = 56%
					list = 42%
					signal = 33%
HALLMARK_PANCREAS_BETA_CELLS	32	−0.54569	−1.65505	0.000911	tags = 41%
					list = 13%
					signal = 35%

### The m6A Risk Score and the Genome Changes in Glioma Patients

**FIGURE 6:**
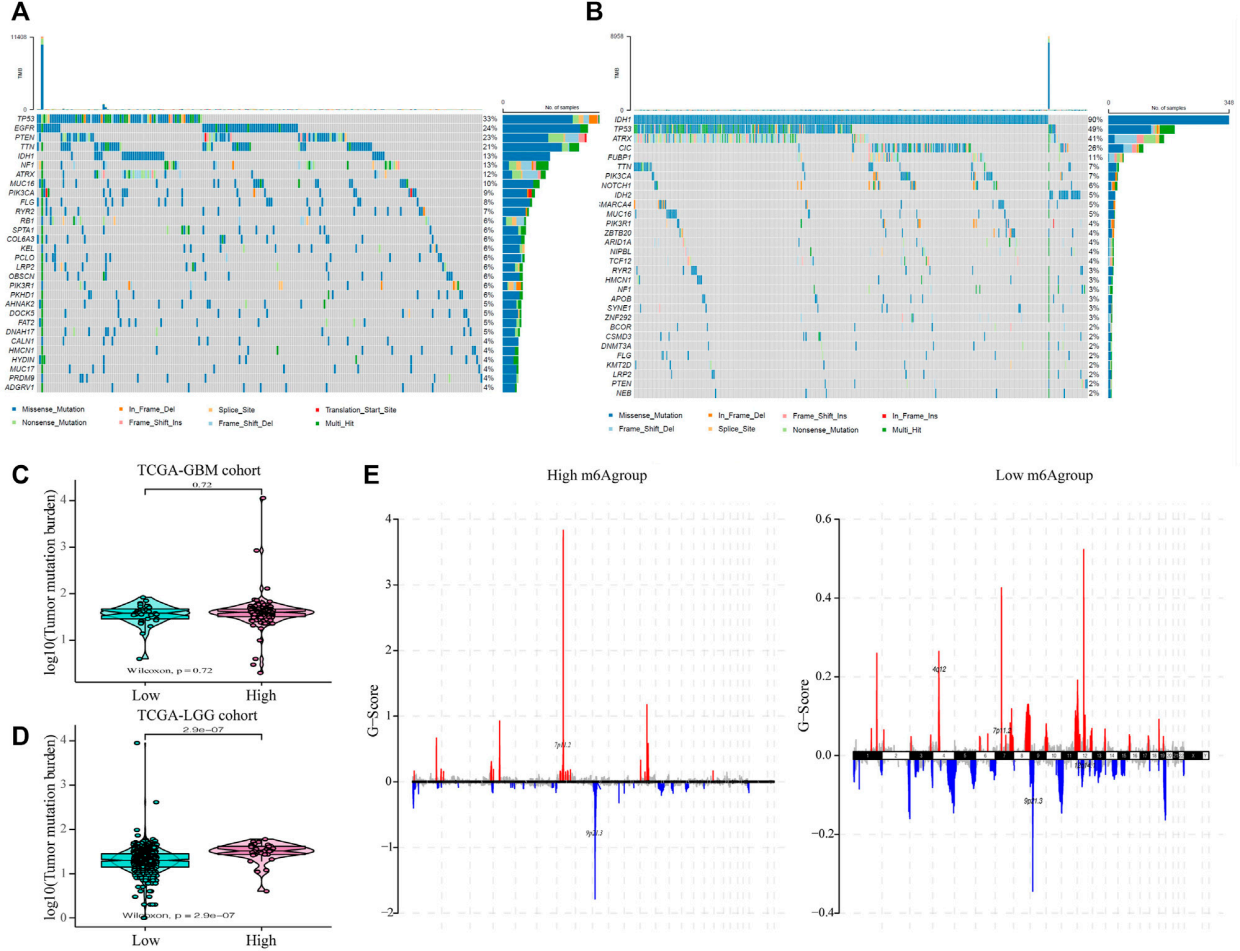
The influence of different m6A risk groups on the genetic variation of GLIOMA patients. **(A–B)** Mutation maps of common tumorigenesis driver genes in glioblastoma multiforme (GBM) and lower-grade glioma (LGG) patients grouped by high and low m6A groups. The mutation information of each gene in each sample is displayed in the waterfall chart with the total percentage of mutation, and various colors indicate different mutation types; the upper section of the legend shows the mutation load; **(C)** Compared with the patients in the low m6A group, there was no significant difference in the tumor mutation level in GBM patients in the high m6A group (*p* = 0.72); **(D)** Compared with patients in the low m6A group, the tumor mutation level in LGG patients in the high m6A group was significantly higher (*p* < 0.001); **(E)** the copy number variation of glioma patients in the high and low m6A groups was different: red indicates an increased copy number, while blue indicates a significant loss in copy number.

Subsequently, we evaluated the effect of the m6A risk score on the genetic variation in glioma patients, including SNPs and copy number variations (CNVs). The single nucleotide mutation analysis of driver genes in common tumorigenesis showed that their SNP levels differed between the high and low groups (Figures 6A,B). At the same time, the overall analysis showed that there was no significant difference in the tumor mutation burden (TMB) between the high and low m6A groups of GBM patients (*p* = 0.73; Figure 6C), while there was a significant difference in the LGG patient group (*p* < 0.001; Figure 6D). Moreover, research on the frequency of CNV changes showed that in patients in the high m6A group, the CNV changes were mainly in the deletion of gene copy numbers. In contrast, in patients in the low m6A group, they reflected gene amplification (Figure 6E).

### The m6A Risk Score and the Immune Characteristics of Glioma Patients

**FIGURE 7:**
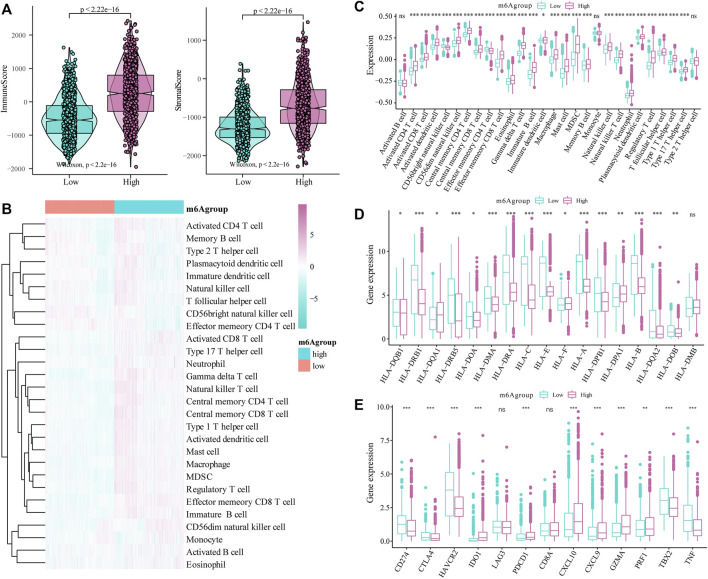
Correlation of the m6A risk score with different immune cell infiltration. **(A)** Compared with the low expression group, the immune-related scores and stromal-related scores of patients in the high-risk group were significantly increased (*p* < 0.001); **(B)** The overall immune infiltration level of glioma patients was analyzed based on the ssGSEA algorithm; **(C)** Correlation analysis shows that there are significant differences in the expression of multiple immune subtypes in patients in the high and low m6A groups; **(D)** There are differences in the expression of multiple HLA family genes between the high and low m6A groups; **(E)** There are also differences in the expression levels of immune therapy-related target genes between the high and low m6A groups.

Next, we evaluated the effect of the m6A risk score on the overall immune characteristics and the different levels of immune cell infiltration in glioma patients. The results showed that compared with the low-risk group, the immune-related and stromal-related scores of patients in the high-risk group were significantly increased (*p* < 0.001, Figure 7A). At the same time, we further used the ssGSEA algorithm to evaluate the infiltration level of 28 different immune cells (Figure 7B). Differential analysis showed that the infiltration levels of multiple immune subgroups were significantly different between the high- and low-risk groups (Figure 7C), including CD8+T cells, activated memory CD4 +T cells, follicular helper T cells, and M1 macrophages. Further analysis showed that the expression levels of multiple HLA family genes and immunotherapy-related targets were significantly different between the high and low m6A groups (Figures 7D,E).

### Analysis of Glioma Patient Sensitivity to Different Small Molecule Drugs Based on the m6A Risk Score

**FIGURE 8:**
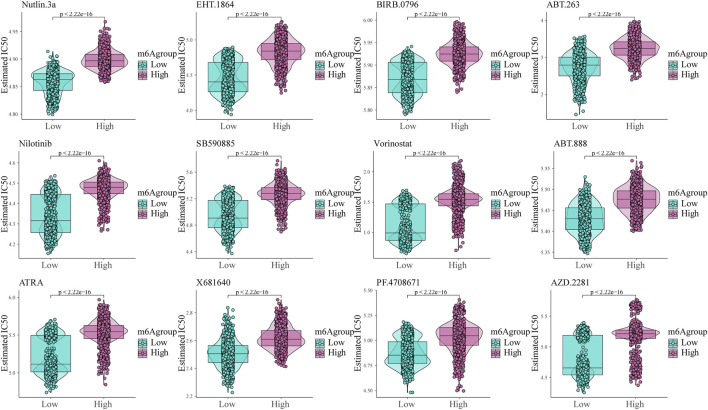
Shows the sensitivity of the m6A risk score to different chemotherapeutics and small-molecule anti-cancer drugs based on the Genomics of Drug Sensitivity in Cancer database.

To analyze glioma patient sensitivity to small molecule drugs based on the m6A risk score, we downloaded the cell line gene mutation data and IC_50_ values of different anti-cancer drugs from the GDSC database. Based on the responsiveness of the cell lines to 138 different chemotherapeutics and small molecule anti-cancer drugs, the IC_50_ values of glioma patients for different drugs were predicted. The results showed significant differences between patients with high and low m6A risk scores (*p* < 0.001; Figure 8), especially Nutlin.3a (p53 activator) (Awan et al., 2021), EHT. 1864 (Rac GTPase inhibitor), (Onesto et al., 2008), and BIRB. 0,796 (pan p38MAPK inhibitor) (Tang et al., 2018).

### Construction of a Clinical Prediction Model Based on the m6A Risk Score

**FIGURE 9:**
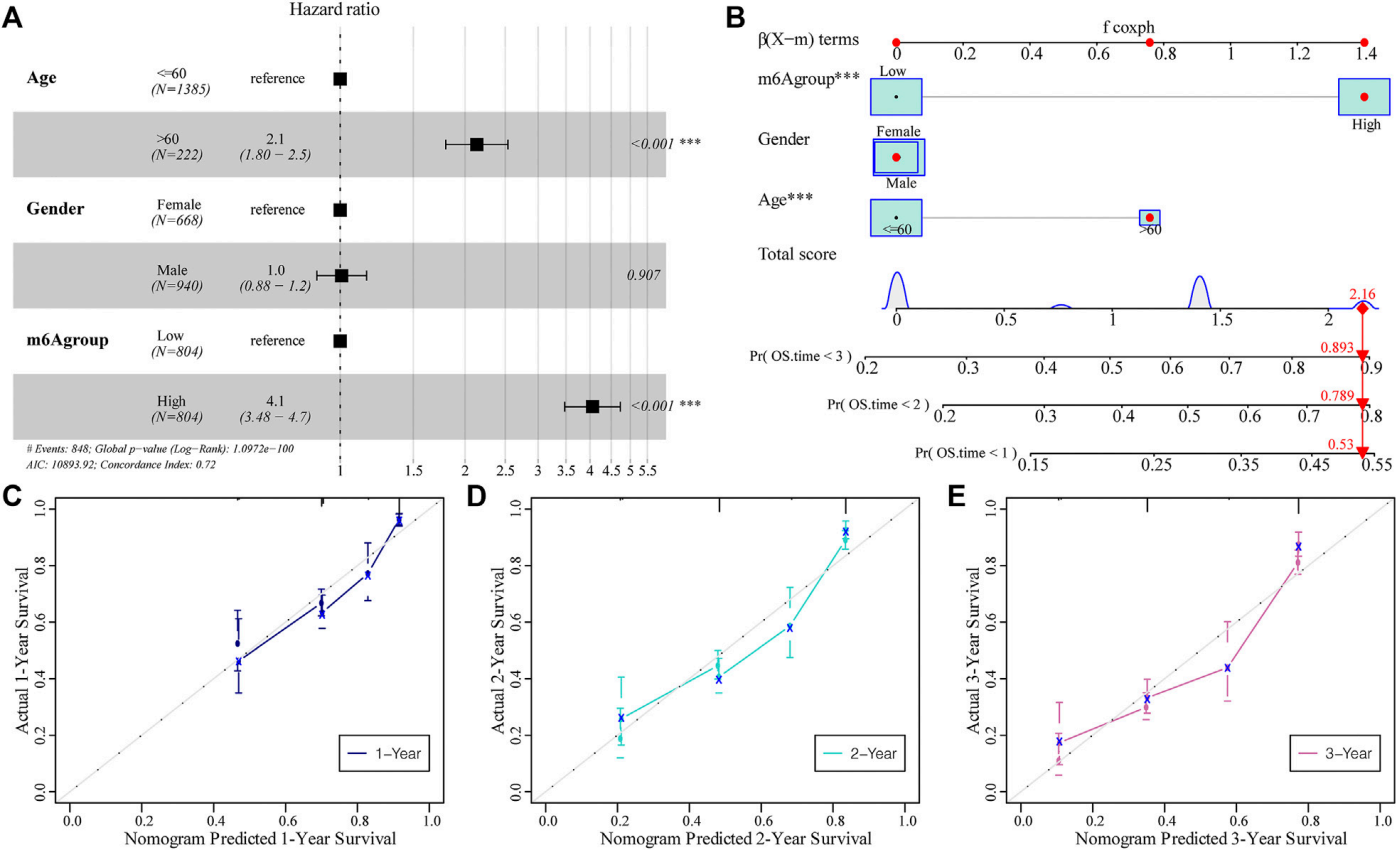
The predictive ability of the m6A risk score on glioma patient prognosis. **(A)** Multivariate Cox regression analysis of risk score combined with clinicopathological characteristics of HR and *p*-values; analysis showed that the m6A group score is an independent risk factor for the prognosis of glioma patients; **(B)** The m6A group score combined with clinicopathological characteristics was selected to construct a clinical prediction model; **(C–E)** The calibration curve of the nomogram; the *x*-axis is the survival predicted by the nomogram, while the *y*-axis is the survival actually observed. The curve shows that the model has a good predictive value for one-, two-, and 3-year predictions.

Subsequently, we assessed the impact of the m6A risk score on the prognosis of patients with glioma. Univariate and multivariate Cox analyses showed that the m6A risk score was an independent risk factor for glioma patient prognosis prediction (Table 4; Figure 9A). The m6A group was combined with different clinicopathological characteristics to construct a nomogram to predict the OS of the patient (Figure 9B). We used the C-Index to calculate the discriminative ability of the nomogram, which showed a high degree of discrimination (0.717 (0.701–0.733)). Moreover, the calibration curve shows that by comparing the one-, two-, and three-year OS estimates, the actual values observed in patients are in agreement (Figures 9C–E).

**TABLE 4:** OS prediction for m6A groups with univariate and multivariate Cox.

	Univariate Cox analysis				Multivariate Cox analysis			
	HR	HR.95L	HR.95H	*p*-Value	HR	HR.95L	HR.95H	*p*-Value
Age (>60 vs ≤60)	2.69	2.26	3.19	1.31E-29	2.14	1.80	2.54	7.12E-18
Gender (Male vs Female)	1.08	0.94	1.24	0.292,437	1.01	0.88	1.16	0.90659
Risk score (High vs Low)	4.29	3.68	5.00	8.30E-78	4.06	3.48	4.73	8.95E-71

### Validation of m6A Gene Expression in Glioma

**FIGURE 10:**
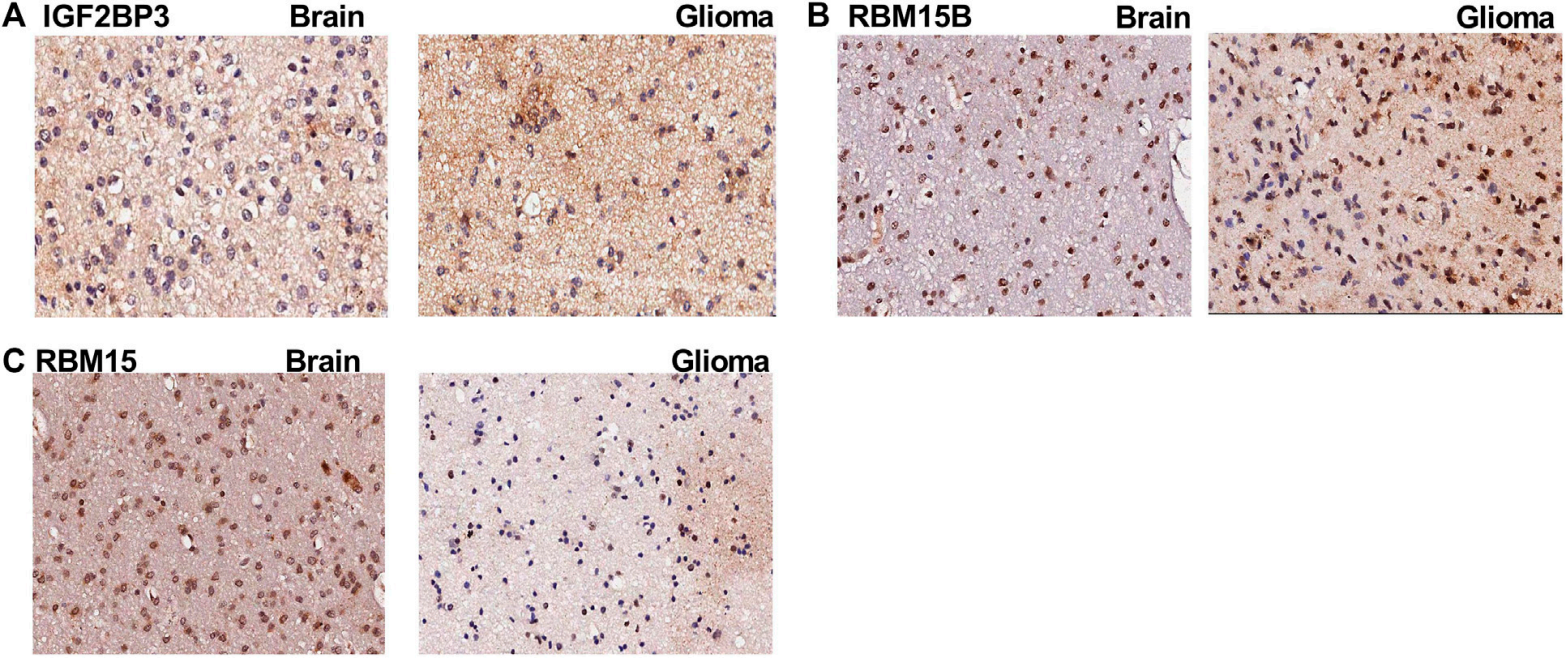
The immunohistochemistry images for m6A genes. **(A)** The immunohistochemistry (IHC) images for IGF2BP3 in para-tumoral and glioma tissue; **(B)** The IHC images for RBM15B in para-tumoral and glioma tissue; **(C)** The IHC images for RBM15 in para-tumoral and glioma tissue.

To validate the expression of m6A genes in glioma, we performed IHC of patient samples. We found that the expression of IGF2BP3 and RBM15B (Figure 10A,B) was increased in the glioma area compared to that in the para-tumor area. In contrast, the expression of RBM15 was obviously reduced in the tumor area (Figure 10C). These findings are consistent with the LASSO Cox model (Table 5).

**TABLE 5:** The Co-efficiency (Coef) for m6A genes.

Gene	Coef
*METTL14*	0.108402272217382
*METTL16*	−0.222287570768418
*RBM15*	0.0522104523971671
*RBM15B*	−0.0030452799684085
*YTHDC1*	0.0454991814409694
*YTHDC2*	0.0519627407645707
*YTHDF2*	0.454471539256783
*HNRNPC*	−0.0875308944826499
*LRPPRC*	−0.546531478124607
*HNRNPA2B1*	0.0343128123862081
*IGFBP1*	−0.128788977174298
*IGFBP2*	0.366833311923007
*IGFBP3*	0.0201841096489989
*RBMX*	−0.0536062665564633
*ALKBH5*	−0.0905278141704579

## Discussion

Glioma is one of the most malignant brain tumors. Currently, no target and treatment strategy is available besides traditional tumor resection, chemotherapy, and radiotherapy to improve the survival status, which might be due to the unclear molecular mechanism (Meyer et al., 2021). To fully understand this, we focused on the most relevant RNA modification, m6A methylation. Our aim was to explore prognosis-related genes and identify m6A-related genes based on the co-expression network. We identified the most prognosis-related m6A genes and classified these glioma patients into m6A high- and m6A low-risk groups using the LASSO regression model. In addition, we identified the representative m6A genes with IHC. We found that the protein expression of IGF2BP3 and RBM15B was increased in the glioma tissue, while the expression of RBM15 was decreased compared to that in the para-tumor area.

To investigate the influence of the m6A risk model on different biological characteristics, we performed GSVA analysis on high- and low-risk groups, used heat maps to show related pathways with significant differential enrichment, and found different pathways, including immune-related features and mismatches. To further clarify the molecular pathology of glioma, we first applied the GO, KEGG, and GSEA methods to assess functional enrichment. From GO and KEGG, we found that the pathways most enriched in DEGs are the synaptic vesicle cycle, insulin secretion, nicotine addiction, GABAergic synapses, relaxin signaling pathway, AGE-RAGE signaling pathway in diabetic complications, ECM-receptor interaction, and protein digestion and absorption. In addition, we used GSEA to evaluate the related biological functions in glioma and found that the most enriched pathways were related to hallmark oxidative phosphorylation, adipogenesis, hedgehog signaling and MYC target V in the low m6A group. The low m6A group had a relatively better survival probability. Therefore, these pathways, especially those of oxidative phosphorylation, adipogenesis, and hedgehog and Myc signaling, may represent novel targets for glioma. Wang et al. recently demonstrated that pharmacologically inhibiting oxidative phosphorylation with NG52 (an inhibitor of phosphoglycerate kinase 1) reduces glioma proliferation both *in vitro* and *in vivo*. NG52 can reduce the production of ATP and ROS in tumor cells and reverse the Warburg effect (Wen-Liang Wang et al., 2021). Cheng et al. found that the knockdown of adipocyte enhancer binding protein 1 (AEBP1) reduces the proliferation, invasion, and apoptosis of human glioma cells. They found that AEBP1 expression is increased in human glioma cell lines and that AEBP1 knockdown reduces the expression of NF-κB (Cheng et al., 2020). Although hedgehog signaling is implicated in cancer and viral infections, its exact role in glioma remains unclear. A recent *in vitro* study showed that naringenin could attenuate glioblastoma cell viability and migration by suppressing the hedgehog signaling pathway (Sargazi et al., 2021); however, to date, no *in vivo* studies have been carried out. Therefore, it would be beneficial to investigate the role of these new targets in the treatment of gliomas in preclinical and clinical studies.

As m6A methylation is a widely present epigenetic modification, we next tried to identify the influence of the m6A risk score on the genomic changes in glioma and found that the SNP levels of driver genes were different between the high and low m6A groups. The CNVs in the two groups were also found to be different. In patients in the high m6A group, the CNV changes were mainly due to the gene copy number deletion, while in patients in the low m6A group these changes were mainly reflected by gene amplification. This indicates that m6A methylation might change CNV in gliomas. However, no study has investigated the relationship between m6A methylation and CNV in gliomas, and this needs to be addressed in future studies.

We further developed a nomogram to predict the survival of patients with glioma based on a series of molecular markers and clinical features. Using univariate and multivariate Cox analyses, we found that older age and m6A risk score were independent risk factors for predicting the prognosis of patients with glioma. A previous study found that age was an independent risk factor for GBM, and aging resulted in a poorer prognosis (Wei et al., 2020). Our results are consistent with those of previous studies. The observed OS at one, two, and 3 years was consistent with the predicted values based on the calibration plot. Therefore, our nomogram could be a good model for clinical practice.

Currently, immune infiltration in brain cancer, especially gliomas, is important in determining treatment strategies. Therefore, we analyzed the correlation between m6A risk scores and different immune cell infiltrations. The results showed that, compared with the low-risk group, the immune-related and stromal-related scores of patients in the high-risk group were significantly increased. We used the ssGSEA algorithm to evaluate the infiltration levels of 28 different immune cells. Differential analysis showed that the infiltration levels of multiple immune subgroups, including CD8+T cells, activated memory CD4 +T cells, follicular helper T cells, and M1 macrophages, were significantly different between the high- and low-risk groups. After setting a statistical threshold, we found that the expression of both HLA family members and immune therapy-related genes was different in the high and low m6A groups. The low m6A group had a higher expression of CD274, CTLA4, HAVCR2, TBX2, and TNF. The high m6A group showed higher expression levels of IDO1, PDCD1, CXCL10, CXCL9, GZMA, and PRF1. This indicates that m6A-related genes might be involved in the immune therapy response in glioma, and this needs to be verified in future studies.

However, some limitations of our study must be addressed. First, to comprehensively clarify the molecular mechanisms underlying the occurrence and development of m6A genes, microarray samples from patients with different stages of glioma are needed. Second, immune infiltration associated with m6A genes remains uncharacterized, and additional investigation between tumor cells and immune cells is necessary to elucidate the biological functions of m6A genes in the glioma immune microenvironment. Third, IDH mutation IDH mutations are among the single most important prognostic factor in gliomas and glioblastomas. The MGMT promoter methylation is also involved in the prognosis in glioma patients, and which is also an important indication for specific therapies. However, we were focusing on the m6A RNA methylation in the current study, other DNA methylation. Nevertheless, it would be very interesting to explore the potential link between DNA methylation and m6A RNA methylation in the current study, other DNA methylation and IDH mutation status was not investigated further in our study. Although both belong to the epigenetic modification, up to now, almost few studies have addressed this. The expression profiles used in our study were obtained from TCGA and CGGA data, which may have led to a batch bias between different datasets. Future external validation of our current findings is needed to verify their clinical application.

In summary, m6A regulatory genes may be reliable biomarkers for glioma patient survival, and the expression of these genes is related to genomic changes. This study may be beneficial for correlating glioma immune cell infiltration and molecular profiling. However, further studies are needed to verify the pathological mechanisms and target these m6A regulatory genes in the clinic as prognostic biomarkers for drug response in patients with glioma.

## Data Availability

The original contributions presented in the study are included in the article/Supplementary Material, further inquiries can be directed to the corresponding authors.
