# Corrigendum: m6A regulatory gene-mediated methylation modification in glioma survival prediction

**DOI:** 10.3389/fgene.2022.1106086

**Published:** 2022-12-05

**Authors:** Guiyun Zhang, Ping Zheng, Yisong Lv, Zhonghua Shi, Fei Shi

**Affiliations:** ^1^ Department of Neurovascular Intervention and Neurosurgery, School of Medicine, Shanghai General Hospital, Shanghai Jiao Tong University, Shanghai, China; ^2^ Department of Neurovascular Intervention, Clinical Center of Neuroscience, Shanghai General Hospital, Shanghai Jiao Tong University School of Medicine, Shanghai, China; ^3^ School of Continuing Education, Shanghai Jiao Tong University, Shanghai, China; ^4^ Department of Neurosurgery, 904th Hospital of PLA, Wuxi, China

**Keywords:** glioblastoma, low grade glioma, TCGA, M6A, immune infiltration

In the published article, there was an error in **Affiliation 1**. Instead of “Department of Neurovascular Intervention and Neurosurgery, School of Medicine, Shanghai General Hospital, Shanghai Jiao Tong University, Shanghai, China”, it should be “Department of Neurovascular Intervention, Clinical Center of Neuroscience, Shanghai General Hospital, Shanghai Jiao Tong University School of Medicine, Shanghai, China”.

The authors apologize for this error and state that this does not change the scientific conclusions of the article in any way. The original article has been updated.

